# Supplementary material for: Tissue-Oxygen-Adaptation of Bone Marrow-Derived Mesenchymal Stromal Cells Enhances Their Immunomodulatory and Pro-Angiogenic Capacity, Resulting in Accelerated Healing of Chemical Burns
Source: Int J Mol Sci. 2023 Feb 17;24(4):4102. doi: 10.3390/ijms24044102 (PMC9963537; doi:10.3390/ijms24044102)
Supplement: Supplementary file 1 [file ijms-24-04102-s001.zip › ijms-2199749-supplementary.pdf]

International Journal of *Molecular Sciences*

Article

Tissue Oxygen Adaptation of Bone Marrow-Derived Mesenchymal Stromal Cells Enhances Their Immunomodulatory and Pro-Angiogenic Capacity, Resulting in Accelerated Healing of Chemical Burns

Supplementary Information

Table S1. Primary eschar desquamation and re-epithelialization rates.

Group	Primary eschar desquamation rate, %			Re-epithelialization rate on day 28, %
	Day 14	Day 23	Day 28	
**Saline**	28,6	71,4	85,7	85,7
**21 % oxygen MSCs**	0	42,9	85,7	85,7
**5 % oxygen MSCs**	14,3	100	100	100

Table S2. Gene-specific primers used in the qPCR experiments.

Gene	Sequence ID	Name of the primer	Sequence of the primer	Product size, bp
IFNG	NM_000619.3	IFNG forward	GGTCATTCAGATGTAGCGGA	281
		IFNG reverse	TGCATCCTTTTTCGCCTTGC	
TNFA	NM_000594.4	TNFA forward	GTTGTAGCAAACCCTCAAGC	148
		TNFA reverse	TCACCCCGAAGTTCAGTAGA	
IL6	NM_000600.5	IL6 forward	ATGTGTGAAAGCAGCAAAGAGG	179
		IL6 reverse	TGCAAGTGCATCATCGTTGT	
IL1B	NM_000576.3	IL1B forward	GATGCACCTGTACGATCACT	229
		IL1B reverse	CACGGGAAAGACACAGGTAG	
IL12A	NM_000882.4	IL12A forward	CTCCTGGACCACCTCAGTTTG	89
		IL12A reverse	AGCTCCCTCTTGTTGTGGAA	
IL23A	NM_016584.3	IL23A forward	CTTCTCTGCTCCCTGATAGC	90
		IL23A reverse	TTCGAAGGATCTTGGAACGG	
IL10	NM_000572.3	IL10 forward	GCAAAACCAAACCACAAGACAG	194
		IL10 reverse	AGTCCTGCATTAAGGAGTCG	
TGFB1	NM_000660.7	TGFB1 forward	TGGTGGAAACCCACAACGAA	113
		TGFB1 reverse	GTAGCGAAGTGCAGGTCAAT	
EGF	NM_001963.6	EGF forward	GGATTGACACAGAAGGAACCA	156
		EGF reverse	TCTATCCACTTCAGGGCTGT	
VEGFA	NM_001025366.3	VEGFA forward	CAGCTACTGCCATCCAATCG	165
		VEGFA reverse	AACACAAGTCCACAGCAGTC	
BFGF	NM_002006.6	BFGF forward	ATGTAGAAGATGTGACGCCG	141
		BFGF reverse	AGTTCGTTTCAGTGCCACAT	
18SRNA	NR_003286.4	18S RNA forward	CAGCCACCCGAGATTGAGCA	253
		18S RNA reverse	GGACAGGACTAGGCGGAACA	

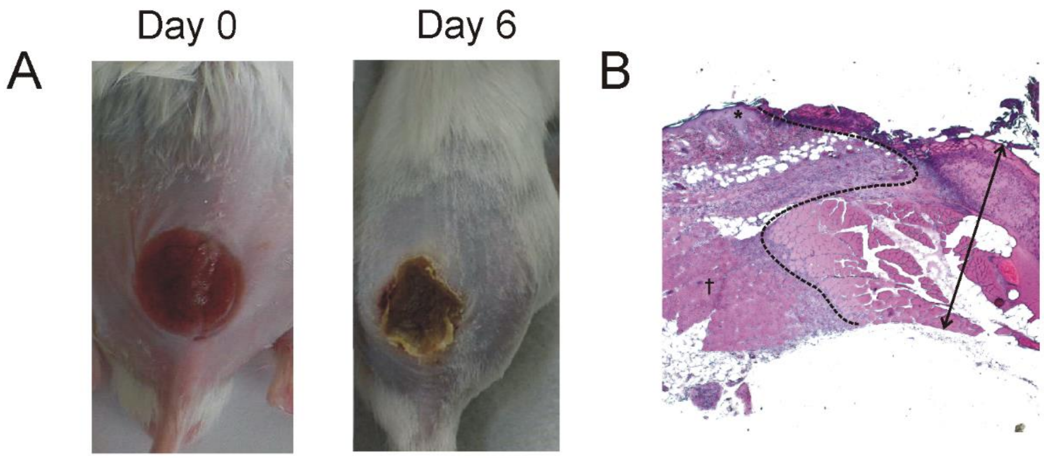

Figure S1. Characteristics of alkali burn wounds on Balb/c mice model. (**A**) Images of the alkali burns immediately after injury and on day 6. (**B**) Image of histological section of the wound and adjacent healthy tissues on day 6 after alkali burn. The border between non-damaged and necrotic tissue is outlined with black dashed line. Double-headed arrow shows full-skin necrosis and necrosis of underlying *panniculus carnosus* (asterisk) muscle. Black star indicates formation of migrating epithelial tongue in the wound edge.

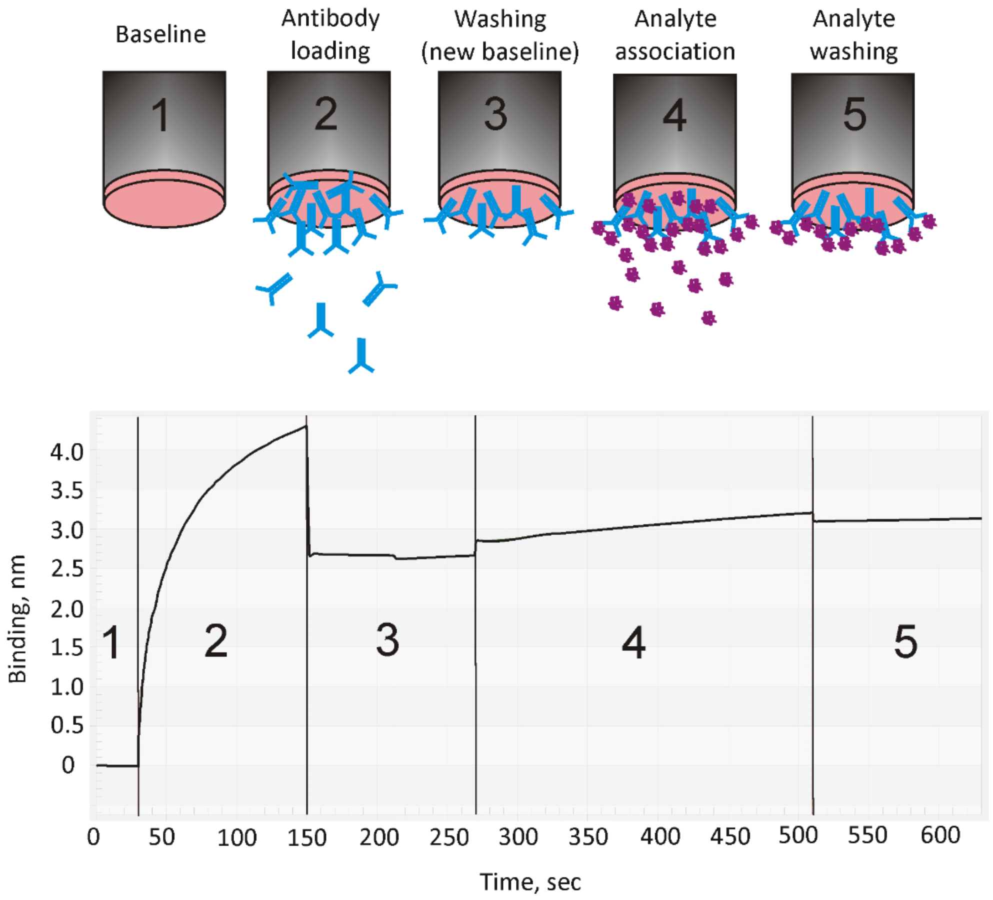

Figure S2. The scheme of bio-layer interferometry measurement of VEGFA binding. The procedure includes 5 steps: 1 – baseline registration (in PBS); 2 – immobilization of antibodies to VEGFA (ab52917, Abcam) on a biosensor with conjugated protein A; 3 – washing out the unbound antibodies and registration of a new baseline; 4 – detection of VEGFA in the sample; 5 – washing out the unbound VEGFA.
